# Assessment of acute flaccid paralysis surveillance performance in East and Southern African countries 2012 - 2019

**DOI:** 10.11604/pamj.2020.36.71.23173

**Published:** 2020-06-08

**Authors:** Daudi Manyanga, Charles Byabamazima, Brine Masvikeni, Fussum Daniel

**Affiliations:** 1 WHO Inter-Country Support Team office for East and Southern Africa, Harare, Zimbabwe

**Keywords:** Polio eradication, acute flaccid paralysis, surveillance, East and Southern Africa, performance indicators

## Abstract

**Introduction:**

polio eradication initiatives started in 1988, this is almost the past 32 years following the WHA resolution 41.8 of eradicating polio by the year 2000. As of 2019, only 3 countries remained to be polio endemic globally, Afghanistan, Pakistan and Nigeria. The east and southern sub-region countries had shown progressive achievement towards polio eradication and to start with the African regional certification. The availability of sensitive AFP surveillance performance is among important strategies in the achievement of polio eradication. We, therefore, decided to conduct this assessment of AFP performance from 2012 to 2019 in the ESA sub-region have evidence documentation and support the certification process of the WHO AFRO region.

**Methods:**

we reviewed all reported acute flaccid paralysis (AFP) cases from 19 countries in the ESA sub region with the date of onset of paralysis from 1 January 2012 to 31 December 2019. The data were run to descriptive analysis based on the personal characteristics and AFP surveillance performance indicators parameters.

**Results:**

a total of 46,014 AFP cases were reported from 19 countries in the ESA countries who were paralyzed from 1 January 2012 to 31 December 2019. The most affected age group was children aged 0 to 3 years old where 19,740 children with acute paralysis were reported representing 42.9% of the total reported AFP for the period. The overall assessment of the non-polio AFP rate, there is an increase from a rate of 2.7 in 2012 to 3.5 in 2019 per 100,000 population aged less than 15 years, reflects a significant change with a p-value of 0.040 (95% C.I. ranges from 0.035 to 1.564). Furthermore, the percentage of stool adequacy raised from 86.4% in 2012 to 88.5% in 2019, with an observed 2.1% difference and no significant change over the 8 years.

**Conclusion:**

we observed an overall increase in the sensitivity of the AFP surveillance performance for the ESA sub-region countries from 2012 to 2019 using the national performance indicators. The COVID-19 pandemic paused an operational challenge for AFP surveillance performances from 2020. A further subnational surveillance performance analysis is suggested.

## Introduction

Polio eradication initiatives started as early as in 1988 when the forty-first world health assembly (WHA) sat in Geneva, from 2 to 13 May and came up with resolution WHA 41.8 for global polio eradication by the year 2000 [1]. The milestones for poliomyelitis eradication were initially revised in 2002 and further later in 2012 to accommodate unexpected challenges which were met on the implementation process [2, 3]. Following the revision of milestones, tremendous efforts towards polio eradication were made. Even though it is over past 30 years ago, the incidence of polio has dropped by more than 99.99%, from about 350,000 cases a year in 125 countries yet there were 175 paralytic wild polio-virus type 1(WPV1) in 2019 from Afghanistan and Pakistan and three remaining polio-endemic countries: Afghanistan, Nigeria and Pakistan [4, 5]. The last country in East and Southern Africa to report paralytic polio caused by wild polioviruses was Ethiopia in 2014 from importation [6]. Though significant progress has been made and observed over years, the last mile of polio eradication seems to be hardest especially since 2018 where paralytic polio cases increased from 22 in 2017 to 33 in 2018 and 175 in 2019 coupled with insecurity challenges, ebola outbreaks and COVID-19 pandemic [5, 7, 8].

The three polio endemic countries, Afghanistan, Nigeria and Pakistan they are all challenged by insecurity and some areas are not reached by immunization and surveillance programs. Furthermore, extensive environmental surveillance have been initiated around the world to supplement AFP surveillance and in the East and Southern Africa sub region where by December 2019 was already being implemented in 9 countries [9, 10]. The use of adaptive innovative surveillance strategies such as electronic surveillance and geographical information system platforms provided surveillance information and intelligence against security threats and made polio eradication easier, more accountable, more focused/targeted and effective than in the previous years, studies evidence in Nigeria, Liberia, Papua New Guinea and other many countries to mention few [11-14]. The available evidence is supporting that poliomyelitis will remain to be the second disease to be eradicated globally even though the pathway had more programmatic and strategic program environment huddles than it was for smallpox eradication.

The African region has been free of wild poliovirus for over three years period and therefore is progressing towards regional certification. Nevertheless, the emerging of circulating vaccines derived poliovirus (cVDPV) type 2 and 3 in the 14 countries of the 47 in the region including countries with accessibility challenges especially security compromised areas. This wave of cVDPV epidemics, in 2018 and 2019, is occurred in the high-risk countries of the African region which include almost half of the target population including Nigeria, DRC, Angola, Cameroon, Niger, Ethiopia, Mozambique, Kenya, Ghana, CAR Benin, Togo and Cote d'Ivoire [5, 15-17]. Only one of the twenty countries in East and Southern Africa had a less sensitive surveillance system to convince the African regional certification commission to accept their polio-free claim certification documentation before the end of 2019. This was contributed by the continuous sub-optimal AFP surveillance performance in the country mainly because of the security challenges, poor infrastructure and weak/fragile health systems.

Nonetheless, tremendous progress has been observed with the implementation of adaptive surveillance strategies to improve the situation. Irrespective of having sufficient information and best practices to share yet there is limited systematic AFP surveillance performance documentation in the ESA sub-region. We, therefore, decided to conduct this assessment of AFP performance in ESA sub-region for the necessary evidenced documentation and may also be used to support the certification process of the WHO AFRO region. This AFP surveillance documentation will also provide additional information for decision-makers and partners on the AFP surveillance for the improved actions especially in the important last mile for polio eradication.

## Methods

**Study area:** the World Health Organization (WHO) East and Southern Africa (ESA) sub-region comprises of 20 countries namely Botswana, Comoros, Eritrea, Ethiopia, Eswatini, Kenya, Lesotho, Madagascar, Malawi, Mauritius, Mozambique, Namibia, Rwanda, Seychelles, South Africa, South Sudan, Tanzania, Uganda, Zambia and Zimbabwe. The sub-region is estimated to have a total population of 459.4 million and 187.5 million under 15 years of age by 2019 [18].

**Study design:** we conducted a retrospective descriptive quantitative study to assess the acute flaccid paralysis (AFP) surveillance performance based on the secondary data submitted in ESA on weekly basis from 2012 to 2019 from the 20 countries in ESA WHO sub-region countries using the main AFP surveillance indicators including the personal characteristics of reported AFP cases, non-polio AFP cases detection rates, percentage of stool adequacy and timeliness in the detection, investigation and stool specimens shipment to the respective polio laboratories. The data was gathered from countries data submitted to WHO for the AFP database from 2012 to 2019. The data can are available in the WHO in the immunization monitoring disease incidences.

**Study subjects:** all investigated and reported AFP patients who were aged less than 15 years of age or whoever patient with paralysis of any age where a clinician suspect poliomyelitis case in the ESA sub-region with the date of onset of paralysis from 1 January 2012 to 31 December 2019.

**Clinical and laboratory investigation:** a WHO standard case definition was used for inclusion and exclusion of AFP cases reported from all countries, regardless of variability in the AFP case investigation occurred across the ESA sub-region from prescribing nurses to clinicians of different qualifications based on the syndromic approach [19, 20]. Nevertheless, validation of reported AFP cases was reported to be done by surveillance officers [21]. In every reported AFP case, two stool specimens were collected with an interval of 24 to 48 hours apart and being transported in the reverse cold chain to the seven WHO accredited polio laboratories for virus isolation in the ESA sub region [22, 23].

**Data collection and analysis:** we retrieved AFP data from 2012 to 2018 from the WHO AFP database archives in ESA sub region. All data set were merged and exported to microsoft access for analysis. EPI info version 3.5.4 was used to run descriptive analysis and supplemented by statistical package for the social sciences (SPSS) version 22 for inferential statistics whenever necessary. Seychelles was excluded in the analysis because, the country did not report any AFP case for the study period 1 January 2012 to 31 December 2019.

## Results

A total of 46,014 acute flaccid paralyzes cases were reported from 19 countries in the East and Southern African countries who were paralyzed from 1 January 2012 to 31 December 2019. The majority (55%) of the reported paralyzed people were men, peaked in 2015 where 57% of the reported AFP were men and women were only 43%. Even though of the slightly observed sex variation between men and women of the reported AFP cases in percentage there is no statistical significance, a p-value of 0.99 (95% C.I. ranges from -10.02 to 9.98). The most age group reported to present as AFP was children aged 0 to 3 years old where 19,740 children with acute paralysis were reported representing 42.9% of the total reported AFP for the period (Table 1). The mean age with AFP for the period was 5.79 years with standard deviation of +5.17 years. Few adults (1.8%) were also investigated as AFP based on the medical clinical description of presenting symptoms of poliomyelitis disease and 12.2% of the reported AFP their age was not recorded in the reviewed database. Regarding the symptoms, the commonest presentation was the lower limbs paralysis representing 84% (ranges from 76.4% to 89.2%) of the total reported AFP for the period, and 75.8% (ranges from 72.6% to 78.5%) presented with fever.

The asymmetrical paralysis occurred in 4% (ranges from 1% to 8%) of the reported AFP cases over the period. In the 8 years of AFP surveillance evaluation, many AFP cases were reported in 2016 (6,537 cases) and few were reported in 2013 (4,624 cases). We also found out, three countries Kenya, Lesotho and Malawi had a low average of non-polio AFP rate of less than 2.5 per 100,000 population aged less than 15 years compared to the other remaining 16 countries for the period between 2012 to 2019 (Table 2). However, further examination of the trend there is was performance declining trend on the non-polio AFP rate in Madagascar, Malawi, Rwanda, Tanzania, Uganda, Zambia and Zimbabwe. In terms of the percentage of stool adequacy, the least performing countries (had an average of less than 80% stool adequacy) for the 8 years were South Africa (67.7%), Botswana (69.4%), Namibia (78.4%) and Malawi (79.4%). For the trend analysis, countries showing a declining trend for the percentage of stool adequacy were Botswana, Eritrea, Mozambique, Namibia, Zambia and Zimbabwe. The peak performance for the non-polio AFP rate in the ESA sub-region for the evaluation period was in 2017 (rate of 4.2 per 100,000 population aged less than 15 years) while for the percentage of stool adequacy was in 2019 (88.5%).

**Table 1:** characteristics (parameters) of the reported acute flaccid paralysis cases and suspected poliomyelitis cases, 2012- 2019, in East and Southern Africa countries

Parameters	2012	2013	2014	2015	2016	2017	2018	2019	Total / Average
# of reported AFP cases	4,971	4,624	4,967	5,811	6,537	6,370	6,475	6,259	46,014
% Male (n=16,537)	54	54	56	57	55	54	55	55	55.0
% Female (n=13,346)	46	46	44	43	45	46	45	45	45.0
% Age 0-3 years (n=19,741)	44.1	43.0	40.8	42.6	44.4	43.6	41.9	42.8	42.9
% Age 4-5 years (n=5,909)	12.5	12.5	13.3	14.0	13.0	12.4	12.5	12.6	12.8
% Age 6-9 years (n=7,707)	16.8	16.7	18.1	17.0	15.9	16.7	16.4	16.3	16.7
% Age 10-14 years (n=6,224)	13.2	14.1	13.1	12.8	13.4	13.3	14.4	13.9	13.5
% Age 15+ years (n=826)	1.6	2.1	2.0	2.0	1.5	1.5	2.0	1.7	1.8
% Age unknown (n= 5,607)	11.8	11.6	12.7	11.6	11.8	12.5	12.7	12.8	12.2
% Fever at onset (n=34,856)	78.5	75.7	76	77.3	78.1	75.1	72.7	72.6	75.8
% Asymmetrical paralysis (n=1,829)	1.0	1.8	4.0	4.0	4.0	4.0	8.0	5.0	4.0
Lower limb paralysis (n=38,847)	88.9	88.5	85.1	76.4	86.3	89.2	81	80	84.4
% Progression in 3 days (Acute) - n= 46,014	100	100	100	100	100	100	100	100	100

**Table 2:** the performances of acute flaccid paralysis surveillance core indicators by countries, 2012-2019, East and Southern Africa

Country	Indicator	2012	2013	2014	2015	2016	2017	2018	2019	Mean
Botswana	NP-AFP rate	3.3	2.4	2.9	3.0	1.8	2.1	2.6	3.1	2.6
	% of stool adequacy	52.0	73.0	50.0	86.0	57.0	80.0	94.7	62.5	69.4
Comoros	NP-AFP rate	1.3	1.5	0.3	0.6	1.7	11.7	0.8	2.7	2.6
	% of stool adequacy	100.0	60.0	100.0	67.0	83.0	93.0	100.0	100.0	87.9
Eritrea	NP-AFP rate	1.4	0.7	0.5	0.8	5.9	7.0	7.0	6.7	3.7
	% of stool adequacy	96.0	100.0	100.0	100.0	97.0	96.0	94.7	89.1	96.6
Ethiopia	NP-AFP rate	2.9	2.4	2.4	2.9	2.6	2.6	2.6	2.9	2.7
	% of stool adequacy	90.0	88.0	86.0	92.0	91.0	91.0	91.8	90.2	90.0
Kenya	NP-AFP rate	0.4	0.3	0.3	3.5	2.9	2.4	3.2	2.8	2.0
	% of stool adequacy	86.0	84.0	81.0	83.0	89.0	82.0	85.8	91.7	85.3
Lesotho	NP-AFP rate	1.8	1.7	3.3	1.9	2.4	1.1	2.2	2.4	2.1
	% of stool adequacy	83.0	83.0	91.0	100.0	100.0	100.0	85.7	100.0	92.8
Madagascar	NP-AFP rate	3.3	3.1	3.3	4.7	7.3	6.3	5.6	5.4	4.9
	% of stool adequacy	86.0	84.0	85.0	61.0	85.0	93.0	94.9	93.9	85.4
Malawi	NP-AFP rate	2.3	2.1	1.1	1.5	2.4	3.7	2.5	2.2	2.2
	% of stool adequacy	79.0	79.0	73.0	80.0	64.0	84.0	87.0	89.4	79.4
Mauritius	NP-AFP rate	2.2	1.9	1.5	2.0	2.8	3.5	3.1	3.1	2.5
	% of stool adequacy	100.0	80.0	100.0	100.0	71.0	63.0	100.0	100.0	89.3
Mozambique	NP-AFP rate	2.8	2.7	2.5	2.2	3.1	3.1	3.6	4.1	3.0
	% of stool adequacy	87.0	88.0	89.0	80.0	81.0	85.0	87.6	72.7	83.8
Namibia	NP-AFP rate	3.9	2.0	4.6	2.8	3.4	2.8	2.2	2.4	3.0
	% of stool adequacy	79.0	89.0	71.0	78.0	87.0	84.0	60.0	79.2	78.4
Rwanda	NP-AFP rate	3.4	3.2	5.3	5.9	4.0	3.0	3.1	2.7	3.8
	% of stool adequacy	98.0	99.0	98.0	98.0	93.0	96.0	87.7	88.5	94.8
South Sudan	NP-AFP rate	4.0	4.3	2.8	3.9	5.9	6.6	7.4	6.3	5.1
	% of stool adequacy	96.0	94.0	93.0	94.0	89.0	83.0	83.0	89.6	90.2
South Africa	NP-AFP rate	2.5	3.6	2.4	3.3	3.2	3.7	3.0	3.7	3.2
	% of stool adequacy	60.0	69.0	63.0	67.0	71.0	64.0	65.2	82.5	67.7
Eswatini	NP-AFP rate	4.3	6.4	3.5	2.8	3.3	3.5	5.1	4.1	4.1
	% of stool adequacy	100.0	89.0	87.0	92.0	79.0	100.0	93.3	94.1	91.8
Tanzania	NP-AFP rate	2.8	3.3	2.9	3.6	4.5	4.3	3.8	3.6	3.6
	% of stool adequacy	93.0	94.0	93.0	97.0	97.0	97.0	98.3	97.8	95.9
Uganda	NP-AFP rate	2.7	2.7	2.9	3.6	4.5	4.3	4.0	2.8	3.4
	% of stool adequacy	86.0	89.0	91.0	83.0	88.0	87.0	90.0	90.0	88.0
Zambia	NP-AFP rate	3.1	2.9	3.0	3.5	3.5	4.3	2.7	2.8	3.2
	% of stool adequacy	85.0	87.0	94.0	88.0	96.0	89.0	85.9	84.2	88.6
Zimbabwe	NP-AFP rate	3.2	4.0	2.7	3.4	3.7	3.2	3.4	2.6	3.3
	% of stool adequacy	86.0	87.0	86.0	85.0	88.0	88.0	93.4	86.1	87.4
ESA Average	NP-AFP rate	2.9	3.1	2.5	2.9	3.6	4.2	3.6	3.5	3.3
	% of stool adequacy	86.0	85.0	85.8	85.8	84.5	87.1	88.4	88.5	86.4

South Africa achieved the percentage of stool adequacy of more than 80% only once (2019) while Namibia and Botswana achieved three times and Malawi four times for the evaluation period. The overall assessment of the percentage of stool adequacy raised from 86.4% in 2012 to 88.5% in 2019, even though there is an observed 2.1% difference, there is no significant change with a p-value of 0.5691 (95% CI ranges from -5,312 to 9.512) over the 8 years. In terms of the non-polio AFP rate, there is an increase from a rate of 2.7 in 2012 to 3.5 in 2019 per 100,000 population aged less than 15 years, reflects a significant change with a p-value of 0.040 (95% C.I. ranges from 0.035 to 1.564). It was observed that an average of 71.6% of the reported AFP cases were notified within 7 days of onset of paralysis for the period, the lowest was 70.2% in 2013 and 2015 while the highest was 72.5% in 2016 and 2019 (Table 3). The very delayed notification (more than 14 days of onset of paralysis) had an average of 7% (ranges from 6.5% in 2017, 2018 and 2019 to 7.7% in 2015). Missing dates contributed to an average of 3.3% (ranges from 1.9% in 2012 to 5.1 in 2017). In making a comparison for 2012 and 2019 performances, we observed an improvement in the early detection of cases from 71.4% (2012) to 72.5% (2019) of the reported AFP cases for the year.

**Table 3:** timeliness of reported AFP cases from onset of paralysis to the delivery of stool specimens in polio laboratories

Parameters (n)	2012	2013	2014	2015	2016	2017	2018	2019	Mean
**Time interval from onset of paralysis to notification**									
% of reported AFP cases notified < 24 hours (n=8,927)	18.3	18.1	18.9	18.5	19.5	20.8	20.6	20.5	19.4
% of reported AFP cases notified in 2- 3 days (n=11,089)	22.8	23.5	23.2	23.7	25.4	24.4	24.9	24.9	24.1
% of reported AFP cases notified in 4-7 days (n=12,930)	30.3	28.6	29.4	28	27.6	26.8	26.8	27.1	28.1
% of reported AFP cases notified in 8 - 14 days (n=8,329)	19.9	19.4	18.9	17.6	17.6	16.5	17.4	17.4	18.1
% of reported AFP cases notified in 15+ days (n=3,221)	6.8	7.6	7.6	7.7	6.7	6.5	6.5	6.5	7
% of reported AFP cases with missing notification date (n=1,518)	1.9	2.7	2	4.6	3.2	5.1	3.8	3.4	3.3
**Time interval from notification to Investigation**									
% of reported AFP cases investigated < 24 hours of notification (n=37,593)	82.9	81.6	78.6	76.9	81.6	79.9	85.4	87	81.7
% of reported AFP cases investigated in 2- 3 days of notification (n=2,853)	7.1	7.1	7.6	6	5.8	5.1	4.9	5.7	6.2
% of reported AFP cases investigated in 4-7 days of notification (n=1,242)	3.7	3.9	2.6	2.6	2.3	1.7	2.2	2.2	2.7
% of reported AFP cases investigated in 8 - 14 days of notification (n=322)	1.1	1.1	0.8	0.8	0.6	0.4	0.4	0.5	0.7
% of reported AFP cases investigated in 15+ days of notification (n=138)	0.4	0.5	0.3	0.3	0.2	0.2	0.4	0.3	0.3
% of reported AFP cases with missing dates (n=3,865)	4.8	5.9	10.1	13.4	9.5	12.7	6.7	4.4	8.4

The results reflect a 1.1% difference, statistically significant with a p-value of < 0.0001 (95% CI ranges from 0.725 to 1.475) and a standard error of 0.191. We also realized that an average of 81.7% of the reported AFP cases were investigated within 24 hours of notification, ranges from 76.9% in 2015 to 87% in 2019. In making the comparison of 2012 and 2019 performances, there is a 4.1% difference for cases investigated with 24 hours of notification. The results show a significant change in a p-value of less than 0.0001 (95% C.I ranges from 2.846 to 5.353). The very delayed investigation after notification (15 days or more) occurred in 0.3% (ranges from 0.2% in 2016 and 2017 to 0.5% in 2013). Missing dates contributed to an average of 8.4% ranges from 4.4% in 2019 to 13.4% in 2015. It was also shown that an average of 96.2% of all reported AFP cases, the first and second stool samples were collected in an interval of 24 to 48 hours apart, ranging from 95.3% in 2017 to 99% in 2019 (Table 4). Furthermore, it was also noted that an average of 0.6% of all reported AFP cases the first and second stools were collected in 1 day this ranges from 0.2% in 2019 to 0.9% in 2017. We also realized there was a wider interval between the collection of first and second stool samples of 5 days or more in an average of 0.8% of all reported AFP cases ranges from 0.3% in 2019 to 1.2% in 2013.

**Figure 1:**
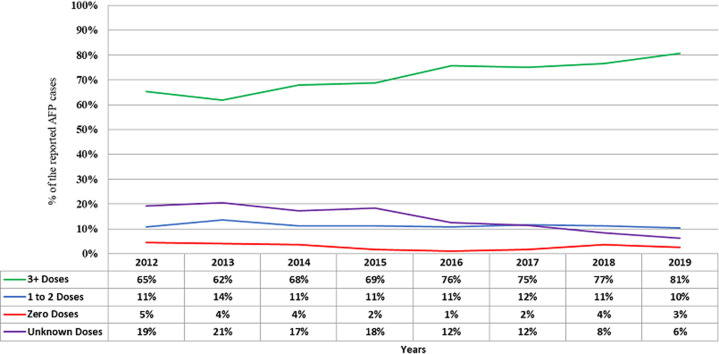
the reported number of OPV doses received by the reported AFP cases from 2012-2019, East and Southern Africa

An average of 1.2% of missing information for dates was also observed for all reported AFP cases ranges from 0% in 2012, 2013 and 2019 to 2.3% in 2015. It was realized that an average of 56.6% of the stool samples collected from the reported AFP cases for the period were delivered in the respective polio laboratories within 72 hours from the collection of second stool samples ranging from 52.8% in 2015 and 2017 to 65.4% in 2012 (Table 4). In making the comparison of 2012 and 2019 performances, there is a decline in the proportion of stool delivered in the laboratory within 3 days after the collection of second stool specimens from 65.4% (2012) to 52.9% (2019). The results indicate a statistically significant change in decline in a p-value of less than 0.0001 (95% C.I ranges from 11.589 to 13.410). However, an average of 13% of stool samples arrived in polio laboratories more than 15 days after the collection of second stool samples (ranging from 7.1% in 2013 to 17.2% in 2016). Furthermore, an average of 9.4% stool samples missed the dates of second stool specimen collection (ranges from 7.4% in 2016 to 14.2% in 2015). We also observed an increase in the number of three or more OPV doses received by the reported AFP cases from 65% (2012) to 81% (2019) indicating a significant difference a p-value of < 0.0001 (95% C.I. ranges from 0.147 to 0.172), the overall average for the period was 71%. Furthermore, we observed a reduction of AFP cases with unknown doses from 19% in 2012 to 6% in 2019 (Figure 1).

**Table 4:** timeliness of stool samples collections the delivery of stool specimens in polio laboratories

Parameters (n)	2012	2013	2014	2015	2016	2017	2018	2019	Mean
**Time interval for first and second stool specimens Collection**									
% of specimens collected < 1-day interval (n =276)	0.7	0.7	0.5	0.7	0.4	0.9	0.5	0.2	0.6
% of specimens collected in 1-day interval (n =41,597)	90.7	91.2	89.6	89.3	90.4	89.8	90.3	92.2	90.4
% of specimens collected in 2 days interval (n=2,669)	5.5	5.8	5.8	5.6	5.4	5.5	5.5	6.8	5.8
% of specimens collected in 3 days interval (n=368)	1.3	0.8	1	0.8	0.8	0.6	0.7	0.4	0.8
% of specimens collected in 4 days interval (n=184)	0.6	0.4	0.5	0.4	0.4	0.3	0.3	0.1	0.4
% of specimens collected in >5 days interval (n=368)	1.1	1.2	0.8	0.9	0.7	1	0.7	0.3	0.8
% of specimens collected with missing dates (n=552)	0	0	1.8	2.3	1.9	2	1.9	0	1.2
**Time interval from the collection of second stool to the delivery of the specimens in polio laboratory**									
% of specimens delivered in lab within 3 days (n=26,044)	65.4	62.7	58.4	52.8	54.3	52.8	53.8	52.9	56.6
% of specimens delivered in lab within 4-10 days (n=7,270)	12.5	17.9	12.3	13.4	16.8	17.1	17.8	18.2	15.8
% of specimens delivered in lab within 11-14 days (n=2,393)	2.5	5.1	3.8	4.9	4.3	6	7.9	7.4	5.2
% of specimens delivered in lab 15+ days (n=5,982)	12.4	7.1	12.7	14.7	17.2	15.6	12.1	12.1	13
% of specimens delivered in lab with missing/Unknown dates (n=4,325)	7.3	7.2	12.7	14.2	7.4	8.5	8.5	9.4	9.4

## Discussion

We observed that over 8 years period, from January 2012 to December 2019 a total of 46,014 AFP cases were reported and all countries reached at least an AFP surveillance certification level in the case detection rate of 1 per 100,000 population aged less than 15 years except Comoros, Eritrea and Kenya. Also, it was noted that from 2016, all countries in ESA reached that certification standard indicating a progressive increase in the sensitivity of AFP surveillance systems. This reflects the results of the efforts, energy and commitment of Governments and the global polio initiative in particular support from the WHO. It is therefore unlikely to have missed cVDPV or WPV circulation in the ESA sub-region countries and can confidently support the certification of the WHO/AFRO region. The study found out the most the reported AFP cases for the period age group was 0 - 3 years which resembles the results from a study in Spain where the majority of cases 310 (45.7%) were under-fives and in Southern China where 75% of patients with AFP were of 0-3 years [24, 25]. The paralysis of the lower-limbs was reported in 84% of the reported AFP cases for the period from 2012 to 2019, this is similar to what was reported in Southern China where 70% of the reported AFP cases had lower-limbs paralysis [25].

The site of paralysis in the AFP cases remained to be an area of concern for the clinician's knowledge because most them only consider paralysis of lower or upper limbs in contrast to the reality that paralysis can occur in any muscle. We, therefore, suggest focused training and sensitization should clearly explain this and consideration to revise AFP cases posters and ensure it includes paralysis of any muscle as defined in the standard case definition. We found out that the overall reported AFP surveillance performances for ESA countries using the national average of two core AFP surveillance indicators, the non-polio AFP rate and percentage of stool adequacy national average are improving especially from 2015. Not surprising, the last case of WPV was reported in 2014 (Ethiopia) for the ESA countries and thereafter a remarkable surveillance performance improvement indicating the is no undetected circulation of either vaccine-derived poliovirus or WPV. Several surveillance strategies have been deployed in the ESA sub region, including peer review planning, use of e-surveillance and others among ESA countries. They all fuel countries' teams to strive for better performances. However, the national averages presented may mask the sub-national surveillance performance gaps which still exist in countries.

This was among this study limitation and therefore we recommend subnational review for countries to be done. A worrisome concern is to the countries who are showing declining for both or either of the one core indicator for the surveillance performances. Projections of AFP surveillance performances to these countries amid to COVID-19 pandemic started in 2019 December indicates possible surveillance and population immunity gaps towards polio eradication. In this regard, post-COVID-19 surveillance and immunization plans are key exits pathways to the challenge in all ESA countries. In terms of notification from the onset of paralysis to the time when the AFP focal person or health facilities focal persons are being notified within 7 days of the cases remained an average of 71.6% indicating early notification to allow two specimens collection within 14 days of onset of paralysis. From the assessment period, we realized an average of 7% were late notification or in other words will lead to directly inadequate stool. However, in further analysis, it is clearly shown notification within 24 hours increased over the period in 2.4% change. This reflects the contribution of the effects of weekly feedback to countries for the improved surveillance decisions, community AFP surveillance and the use of innovative adaptive surveillance strategies such as AVADAR [13, 14].

However, the implementation of AVADAR in some countries remained to be a challenge and may not be an optional strategy to maintain early notification as the strategy is expensive. Furthermore, the system may harm other community surveillance systems because of its motivation benefits which are not embedded in other surveillance systems. We also realized some of the first and second stool specimens were collected within 24 hours in contrary to the guideline that should be in an interval of 24 to 48 hours apart. This is an area again which we understand needs to be well communicated to countries for the quality sensitive surveillance system. Nevertheless, this is among the key AFP surveillance indicators that are being monitored weekly. In this study, it was revealed that AFP stool samples delivery was delayed in reaching the polio laboratories from the collection of second stool samples in the period for an average of 13%. This was thought to contribute negatively to the stool specimens' condition as the result of the reverse cold chain breakdown at any point. We also observed missing data as a challenge for almost all the variables involved in the AFP surveillance operations. This calls for the country's data managers' commitments for the data cleaning.

## Conclusion

We observed an increase in the AFP surveillance performance for the ESA sub region from 2012 to 2019 using the national performance indicators. The review indicates an improvement in the sensitivity of the AFP surveillance system with less likelihood of having missed ongoing transmission of either cVDPV or WPV. The assessment also found out a decline in stool specimen shipment to laboratory over the 8 years period, this needs to be address for further improvement in future. However, subnational surveillance performance gaps may exist and will, therefore, call for a further subnational review. Our findings are suggesting readiness for the ESA sub region AFP surveillance performances support the WHO/AFRO region certification. Also, we have foreseen possible performance decline as an impact of the Covid-19 pandemic.

### What is known about this topic

- Polio endgame strategy 2019-2023 indicates acute flaccid paralysis as, among the key strategies to eradicate poliomyelitis, this is being implemented in East and Southern Africa countries;
- There is no wild poliovirus (indigenous or importation) in East and Southern Africa countries since 2014;
- The East and Southern Africa sub-region WHO office is monitoring AFP surveillance performance indicators to achieve polio eradication.

### What this study adds

- The study identifies weaknesses and progress and recommends ways of improving surveillance performances to achieve regional certification and beyond;
- The study provides valuable information on the trend of acute flaccid paralysis surveillance performance for the ESA countries for eight years compared to Africa regional and global polio surveillance standards;
- The study indicates the need for countries in ESA to focus to the sub-national/district surveillance gaps and good performing countries to avoid complacency in this last mile for polio eradication.
